# Clinical efficacy of AI-navigated transperineal MRI-TRUS fusion biopsy for prostate cancer diagnosis: A retrospective cohort study

**DOI:** 10.1097/MD.0000000000050591

**Published:** 2026-09-18

**Authors:** Jiangchuan Chen, Qiao Xu, Changlong Li, Jiamo Zhang

**Affiliations:** a Department of Urology, Yongchuan Hospital Affiliated to Chongqing Medical University, Chongqing, China; b General Practice School of Chongqing Medical University, Chongqing, China.

**Keywords:** AI fusion biopsy, image registration, mpMRI-TRUS, prostate cancer

## Abstract

This study evaluates the clinical efficacy of transperineal 3D image registration combined with AI-electromagnetic navigation-guided mpMRI-TRUS fusion biopsy versus transrectal cognitive fusion biopsy for prostate cancer diagnosis. This retrospective cohort study analyzed 283 patients undergoing prostate biopsy at Yongchuan Hospital of Chongqing Medical University (January 2022 to December 2024). Participants were stratified into transrectal cognitive fusion (n = 146) and transperineal AI-navigated fusion (n = 137) groups. Detection rates of clinically significant prostate cancer (csPCa) and complication profiles were compared. Multivariable logistic regression identified independent predictors of csPCa detection. The transperineal AI-navigated fusion group demonstrated a significantly higher csPCa detection rate compared to the transrectal cognitive fusion group (55.5% vs 45.2%, *P* = .043). Multivariable logistic regression revealed that advanced age (OR = 1.05, 95% CI: 1.01–1.17), PI-RADS score > 3 (OR = 9.4, 95% CI: 3.95–16.74), and transperineal approach (OR = 3.65, 95% CI: 1.22–4.75) independently predicted csPCa detection. The AI-navigated group showed no hematochezia (0% vs 9.6%, *P* = .003), no sepsis (0% vs 2.1%, *P* = .246), and a significantly lower urinary tract infection rate (13.9% vs 28.8%, *P* = .035). However, urinary retention was significantly more frequent in the transperineal group (14.4% vs 7.3%, *P* = .012), although all cases resolved within 72 hours with conservative management. Transient perineal pain was also more frequent in the transperineal group (24.1% vs 8.9%, *P* = .017). AI-navigated transperineal fusion biopsy was associated with improved csPCa detection and a lower infectious complication rate compared to transrectal cognitive fusion biopsy, but with a significantly higher incidence of transient, self-limiting urinary retention. These findings support its potential as an effective diagnostic strategy, with the caveat that patients should be counseled regarding post-biopsy voiding function.

## 1. Introduction

Prostate cancer (PCa) is a prevalent malignant tumor in males. According to statistical analyses by the American Cancer Society, PCa ranked first in incidence (29%) and second in mortality (11%) among male cancers in the United States in 2023.^[1]^ Globally, approximately 1.2 million new PCa cases are diagnosed annually, with nearly 70% originating from developing countries, posing a significant threat to the health of middle-aged and elderly men.^[2,3]^ The prognosis of PCa primarily depends on the tumor grade and stage at initial diagnosis: patients with early-stage or clinically localized PCa exhibit a 10-year survival rate exceeding 99%, whereas those diagnosed at advanced stages or with distant metastases have a 5-year survival rate of only 30%.^[4]^ Although China demonstrates a lower PCa incidence compared to Western countries, domestic multicenter studies indicate that approximately two-thirds of Chinese patients are diagnosed at intermediate or advanced stages, resulting in a poorer overall prognosis than in Western populations.^[5]^ Therefore, enhancing early screening and diagnostic detection rates for PCa can effectively improve patient prognosis and survival. Improving early diagnosis remains a critical challenge in clinical practice. Prostate needle biopsy serves as the gold standard for PCa diagnosis.^[6]^ Currently, ultrasound-guided transperineal and transrectal systematic biopsy (SB) are the 2 most common methods for obtaining prostate tissue to diagnose PCa.^[7]^ In recent years, the integration of multi-parametric MRI (mpMRI) into prostate cancer (PCa) diagnostic protocols has significantly enhanced lesion characterization through combined T2-weighted imaging, diffusion-weighted sequences, and dynamic contrast enhancement.^[8-11]^ Suspicious lesions identified on mpMRI can be subjected to targeted biopsy, enabling better detection of PCa – particularly clinically significant prostate cancer (csPCa). This advancement has led to the development of targeted biopsy (TB) techniques. Previous studies demonstrate that the combined SB + TB approach, integrating cognitive mpMRI with transrectal ultrasound (TRUS) fusion, achieves superior PCa detection rates compared to SB alone. However, this technique requires substantial expertise due to its technical complexity, steep learning curve, and challenges in detecting satellite microlesions, which may increase the risk of missed diagnoses.^[12]^

With advancements in AI software and image fusion processing, 3D image registration combined with electromagnetic navigation technology has demonstrated potential applications in prostate biopsy.^[13]^ This study aimed to compare the clinical efficacy of 2 mpMRI-TRUS fusion approaches under electromagnetic navigation: AI-based fusion (AI fusion) and cognitive fusion (cognitive mpMRI-TRUS fusion) in prostate biopsy.

## 2. Materials and methods

### 2.1. Research design

A retrospective cohort of 348 patients undergoing prostate biopsy at Yongchuan Hospital of Chongqing Medical University (January 2022 to December 2024) was analyzed. Data were accessed for research purposes on 05/05/2025. Inclusion criteria were: Suspicious nodules detected by digital rectal examination (DRE) with any total prostate-specific antigen (tPSA) level; suspicious lesions identified via transrectal ultrasonography (TRUS) or mpMRI, regardless of PI-RADS (Prostate Imaging Reporting and Data System) category or tPSA level; tPSA > 10 μg/L; tPSA 4–10 μg/L with a free-to-total PSA ratio (f/t PSA) < 0.16 or prostate-specific antigen density (PSAD) > 0.15 ng/mL^2^.^[14,15]^ Exclusion criteria included: Acute infection or febrile conditions; hypertensive crisis; decompensated cardiac insufficiency; coagulopathies; unstable glycemic control in diabetes mellitus; severe hemorrhoids, perianal, or rectal pathologies.^[16,17]^ Allocation was not randomized; the choice of biopsy technique was influenced by clinical factors (e.g., preference for a transperineal approach for anterior lesions) and temporal trends as the AI-navigated system was introduced later in the study period. Based on these real-world selections, participants underwent either transrectal cognitive fusion (n = 146) or AI-navigated transperineal fusion (n = 137).

### 2.2. Ethical approval declarations

This study was approved by the Ethics Committee of Yongchuan Hospital Affiliated with Chongqing Medical University (2025EC0132) and was conducted in accordance with local legislation and institutional requirements. The participants provided their written informed consent to participate in this study. All procedures followed the Declaration of Helsinki.

### 2.3. Baseline characteristics

The following baseline variables were extracted from medical records: Demographics: Age at diagnosis and biopsy-naïve status; serological markers: Prostate-specific antigen (PSA) levels (ng/mL) and prostate volume (mL) via transrectal ultrasound; imaging metrics: PI-RADS v2.1 scores (1–5) and suspicious anterior/posterior zonal involvement; procedural details: Systematic + targeted core numbers.

### 2.4. Integrated imaging protocol

All participants underwent standardized 3.0T multi-parametric MRI (MAGNETOM Skyra, Siemens Healthineers, Germany) with endorectal coil assistance prior to biopsy. MRI was performed within 1 week before biopsy. The protocol encompassed axial/coronal T2-weighted imaging (TR/TE 3800/101 ms, slice thickness 3 mm), diffusion-weighted sequences at *b*-values of 50, 800, and 1500 s/mm^2^ with monoexponentially derived ADC maps, and dynamic contrast-enhanced imaging using 0.1 mmol/kg gadobutrol injected at 3 mL/s. A subspecialty-trained genitourinary radiologist (13 years’ experience) interpreted studies following institutionally modified PI-RADS v2.1 criteria: transition zone lesions requiring ≥1.5 cm^3^ volume for category 3 designation, and ESUR 2021 consensus adjustments for diffusion-weighted scoring in anterior fibromuscular stroma.^[13]^

### 2.5. Perioperative management

All patients received bowel preparation with polyethylene glycol electrolyte powder before dinner on the preoperative day. Intravenous levofloxacin (500 mg) was administered 1 hour preoperatively for infection prophylaxis. Postoperative antibiotic therapy was continued for 3 to 5 days if fever or urinary tract infection occurred. In cases of urinary retention, catheterization with a 16-Fr urinary catheter was performed. Postoperative complications were defined as follows: urinary tract infection as a positive urine culture (≥10^5^ CFU/mL) with at least 1 irritative urinary symptom; sepsis according to Sepsis-3 criteria (suspected infection with an acute increase in SOFA score ≥ 2); and hematuria graded by the Clavien-Dindo classification. All complications were recorded within 30 days post-biopsy.

### 2.6. Biopsy protocol

**Figure 1.**
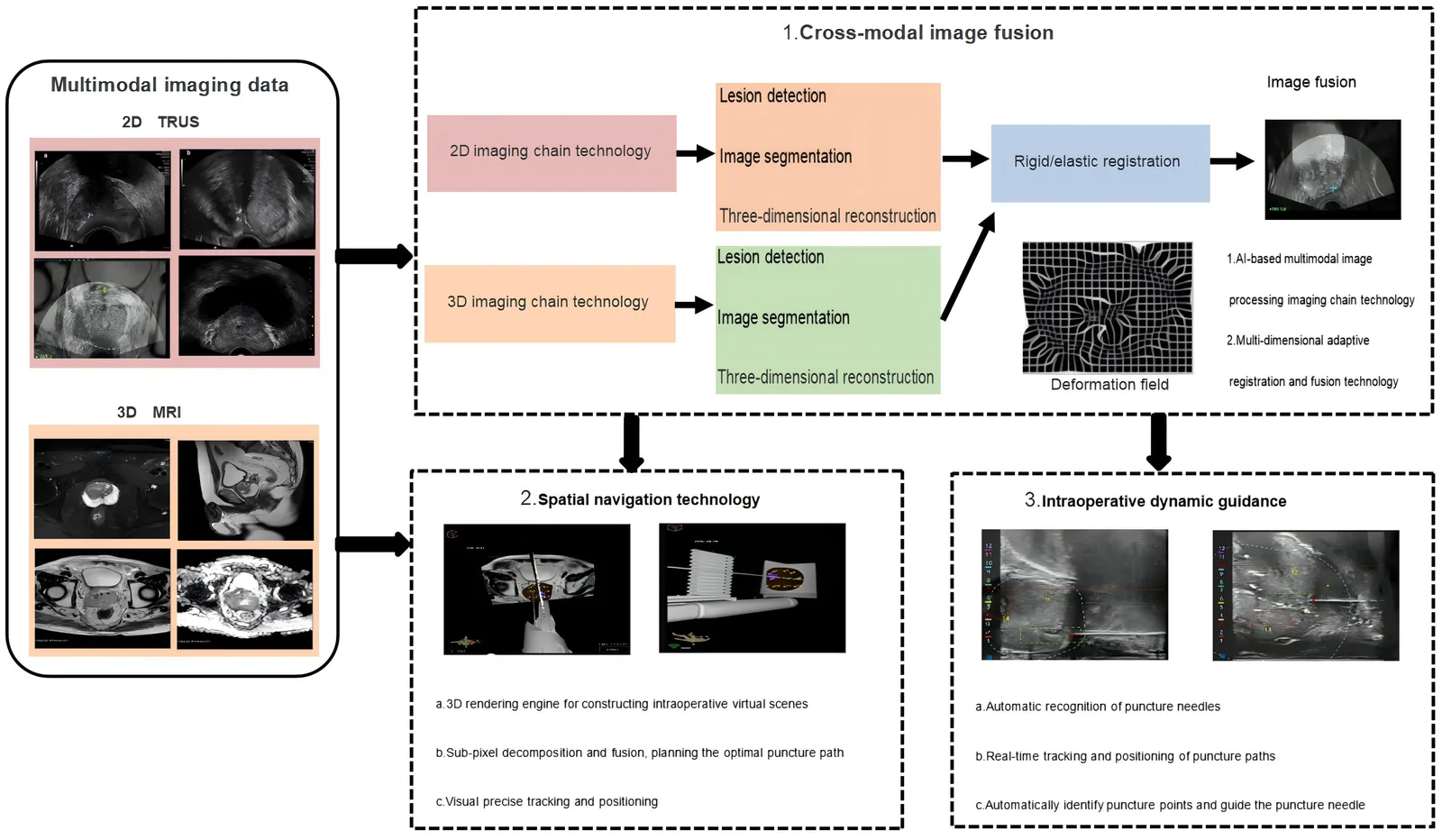
Technical workflow of 3D image registration integrated with AI-electromagnetic navigation. AI = artificial intelligence, MRI = magnetic resonance imaging, TRUS = transrectal ultrasonography.

**Figure 2.**
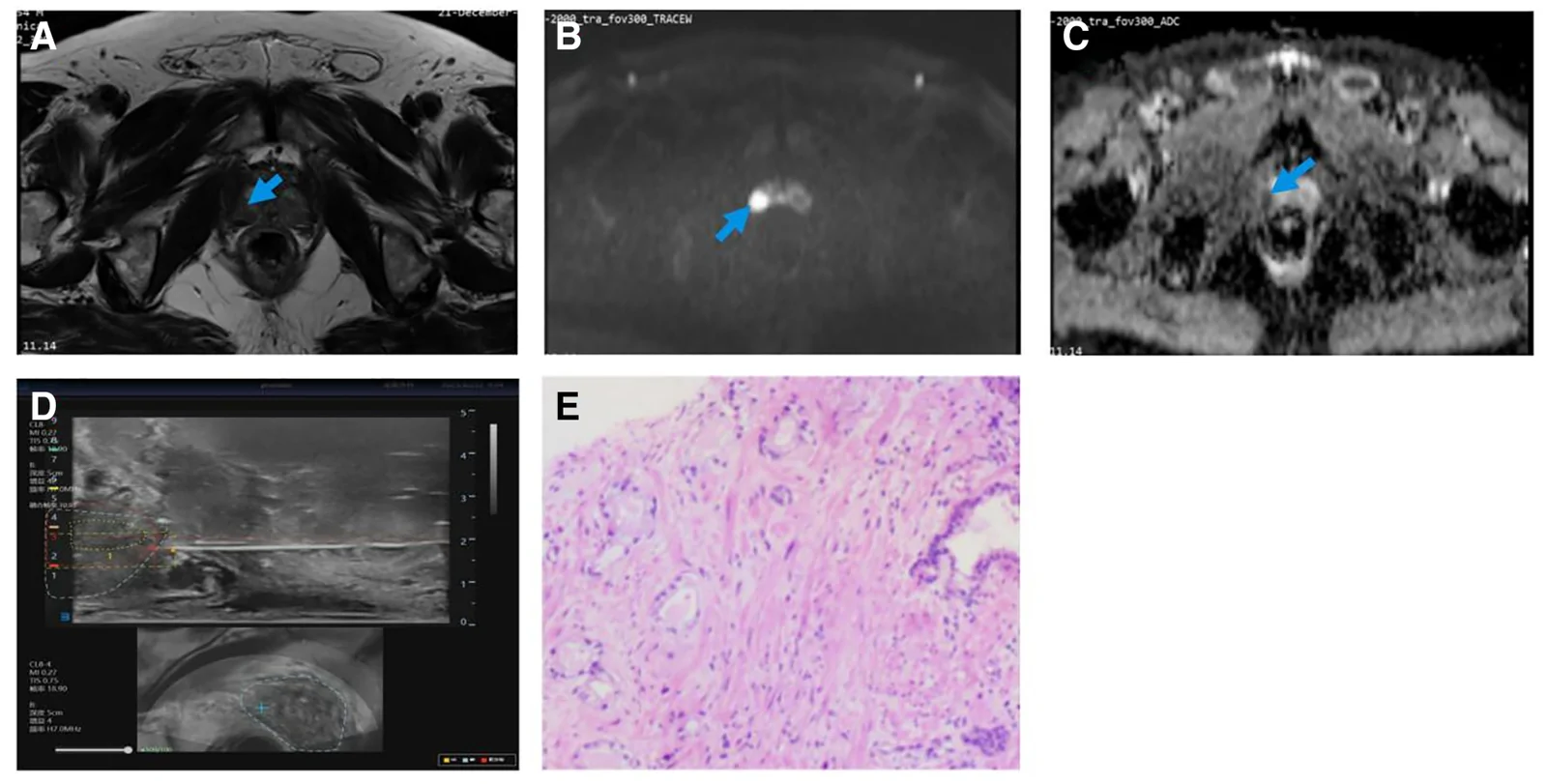
A 65-year-old male undergoing TRUS-mpMRI fusion biopsy (PSA: 11.923 ng/mL, PI-RADS 4). (A) T2-weighted imaging (T2WI) demonstrates heterogeneous high-low signal intensity in the central gland, with a 1.0 × 0.9 cm nodular lesion showing diffuse hypointensity at the 7–8 o’clock position of the peripheral zone. (B) Diffusion-weighted imaging (DWI) reveals hyperintense signal within the lesion. (C) Apparent diffusion coefficient (ADC) map confirms corresponding hypointensity. (D) TRUS-mpMRI fusion imaging precisely localizes the target lesion (arrow). (E) Histopathology confirms prostatic adenocarcinoma with a Gleason score 3 + 4 = 7 [WHO/ISUP] grade 2). ADC = apparent diffusion coefficient, csPCa = clinically significant prostate cancer, DWI = Diffusion-weighted imaging, mpMRI = multi-parametric magnetic resonance imaging, PI-RADS = Prostate Imaging Reporting and Data System, PSA = prostate-specific antigen, PSAD = prostate specific antigen density, T2WI = T2-weighted imaging, WHO/ISUP = World Health Organization/International Society of Urological Pathology.

**Figure 3.**
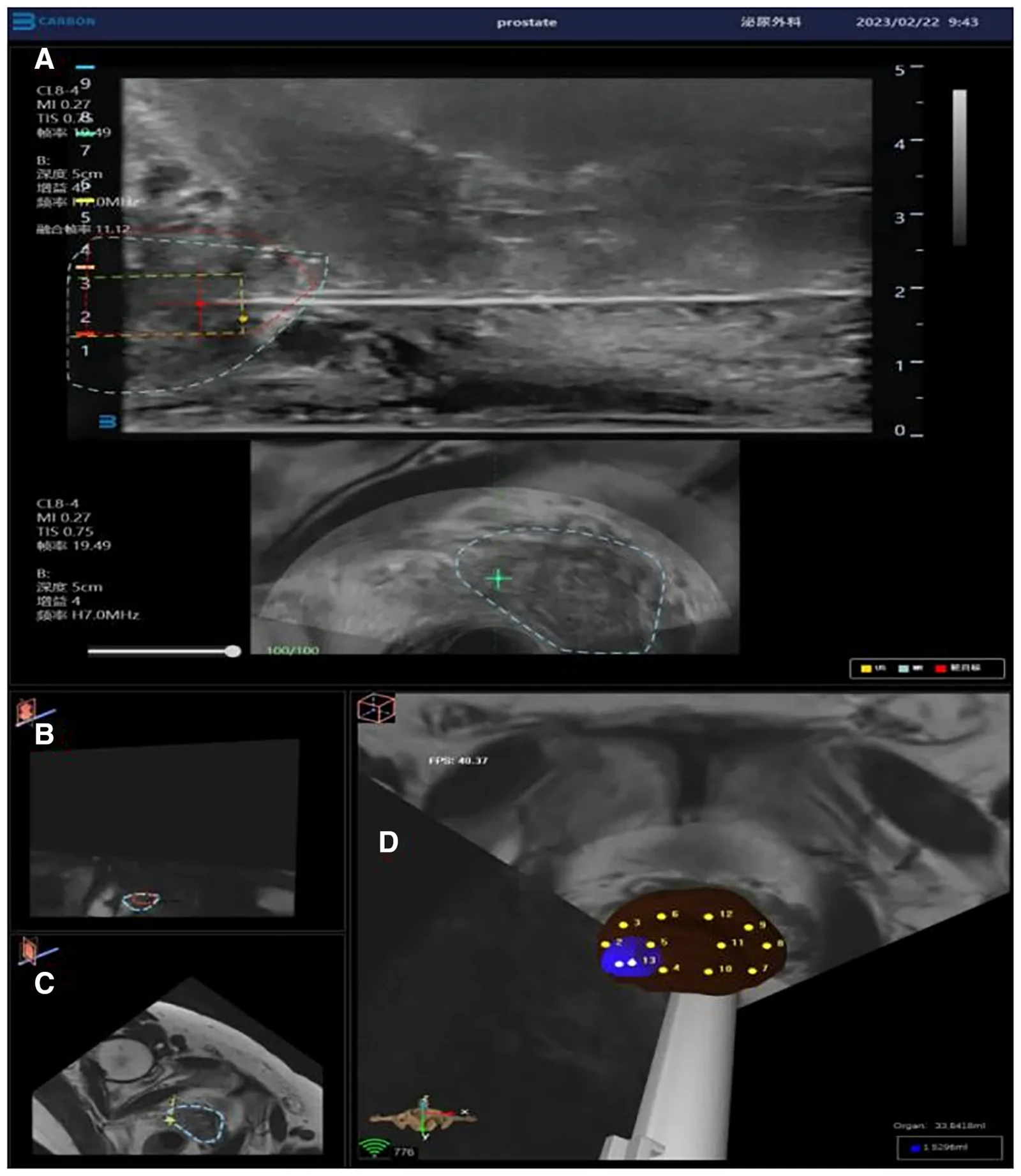
Electromagnetic navigation and needle tip guidance. (A) Target localization criteria and real-time needle tracking within the prostate (arrowheads indicate needle trajectory). (B, C) Three-dimensional (3D) reconstruction of prostate and pelvic structures. (D) Electromagnetic navigation interface demonstrating biopsy targets: systematic sites 1 to 12 (white markers) and targeted site 13 (blue annotation). AI = artificial intelligence, csPCa = clinically significant prostate cancer.

Both groups underwent combined systematic (SB) and targeted biopsies (TB), with SB collecting 12 cores via standard protocols and TB sampling 2 to 3 cores per MRI-visible lesion. All procedures were performed under perineal local anesthesia in a lithotomy position by an experienced urologist. The workflow of the 3D image registration and AI-electromagnetic navigation is illustrated in Figure 1. In the cognitive fusion group, urologists and radiologists collaboratively annotated suspicious lesions on preoperative mpMRI, guiding real-time TRUS adjustments (Capen Medical, Shenzhen) to align with MRI targets for transrectal biopsy. The AI fusion group utilized the VENUS system (Capen Medical, Shenzhen) to fuse mpMRI with real-time TRUS (Fig. 2), enabling template-guided transperineal biopsies (2–3 cores per target + 12-core SB) with needle tracking calibration (Fig. 3). Postoperative outcomes included csPCa detection rates and complications (hematuria, urinary retention, UTI).

### 2.7. Histopathological evaluation

Histopathological evaluation adhered to the 2014 ISUP grading criteria, with clinically significant disease defined as ISUP Grade Group ≥ 2 (Gleason score ≥ 3 + 4 = 7) per 2014 ISUP/2019 WHO criteria. Pathologists were blinded to the biopsy approach.

### 2.8. Statistical analysis

Data processing utilized SPSS 22.0 (IBM Corp, Armonk) with the Advanced Models module. Gaussian-distributed metrics (e.g., PSA levels) were summarized as mean ± SD and analyzed via independent *t* tests, while nonparametric variables (prostate volume) used median with 25th to 75th percentiles and Wilcoxon rank-sum tests. Dichotomous clinical indicators (biopsy approach: transrectal vs transperineal) underwent comparative assessment through χ^2^ tests with Yates’ continuity correction. Clinically significant prostate cancer (csPCa) detection frequencies and post-procedural events were evaluated using Fisher exact test for sparse cells (<5 expected counts). Stratified analyses by PI-RADS categories (per NCCN prostate imaging guidelines) employed Mantel–Haenszel χ^2^ for trend. Multivariable logistic regression was performed to identify independent predictors of csPCa detection. Variables with *P* < .1 in univariable analysis were entered into the model using a backward elimination procedure. Results are reported as odds ratios (OR) with 95% confidence intervals (CI).

## 3. Results

### 3.1. Baseline characteristics

A total of 283 male patients were consecutively enrolled according to the predefined inclusion and exclusion criteria (see Section 2 for details), including 146 patients in the Cognitive Fusion Group and 137 patients in the AI Fusion Group (Table 1). No significant differences were observed between the groups in median age, PSA levels, prostate volume, PI-RADS scores, or total number of biopsy cores (*P* > .05).

**Table 1:** Patient characteristics (n, %).

Variables	Cognitive fusion (n = 146)	AI fusion (n = 137)	*P*
Age, yr ($\bar{x}$±*s*)	62.67 ± 5.94	64.17 ± 6.63	.643
PSAD	0.18 (0.12–0.33)	0.18 (0.13–0.37)	.792
Core length, mm	12.4 (8.43–14.7)	14.5 (11.42–18.4)	.219
Prostate volume, mL	47.62 (33.43–58.92)	49.55 (35.8–61.52)	.533
tPSA, ng/mL	11.43 (7.55–16.28)	10.64 (7.72–15.82)	.638
PI-RADS, n (%)			.417
1–2	12 (8.2)	10 (7.3)	
3	24 (16.4)	26 (19.0)	
4	63 (43.2)	61 (44.5)	
5	47 (32.2)	40 (29.2)	
TB + SB core number, n	16 (16–22)	17 (18–25)	.836
TB, n	3 (2–5)	4 (2–6)	
SB, n	12 (10–13)	12 (10–14)	
Target lesion location, n (%)			.458
Anterior	52 (35.6)	51 (37.2)	
Posterior	94 (64.4)	86 (62.8)	
DRE, n (%)			.622
Positive	115 (78.8)	104 (75.1)	
Negative	31 (21.2)	34 (24.9)	
Prior biopsy status, n (%)			.586
Biopsy-naive, n (%)	135 (92.5)	124 (90.5)	
Repeat biopsy, n (%)	11 (7.5)	13 (9.5)	

DRE = digital rectal examination, PI-RADS = Prostate Imaging Reporting and Data System, PSAD = prostate specific antigen density, SB = systematic biopsy, TB = targeted biopsy, tPSA = total prostate-specific antigen.

### 3.2. Comparison of csPCa detection rates

**Figure 4.**
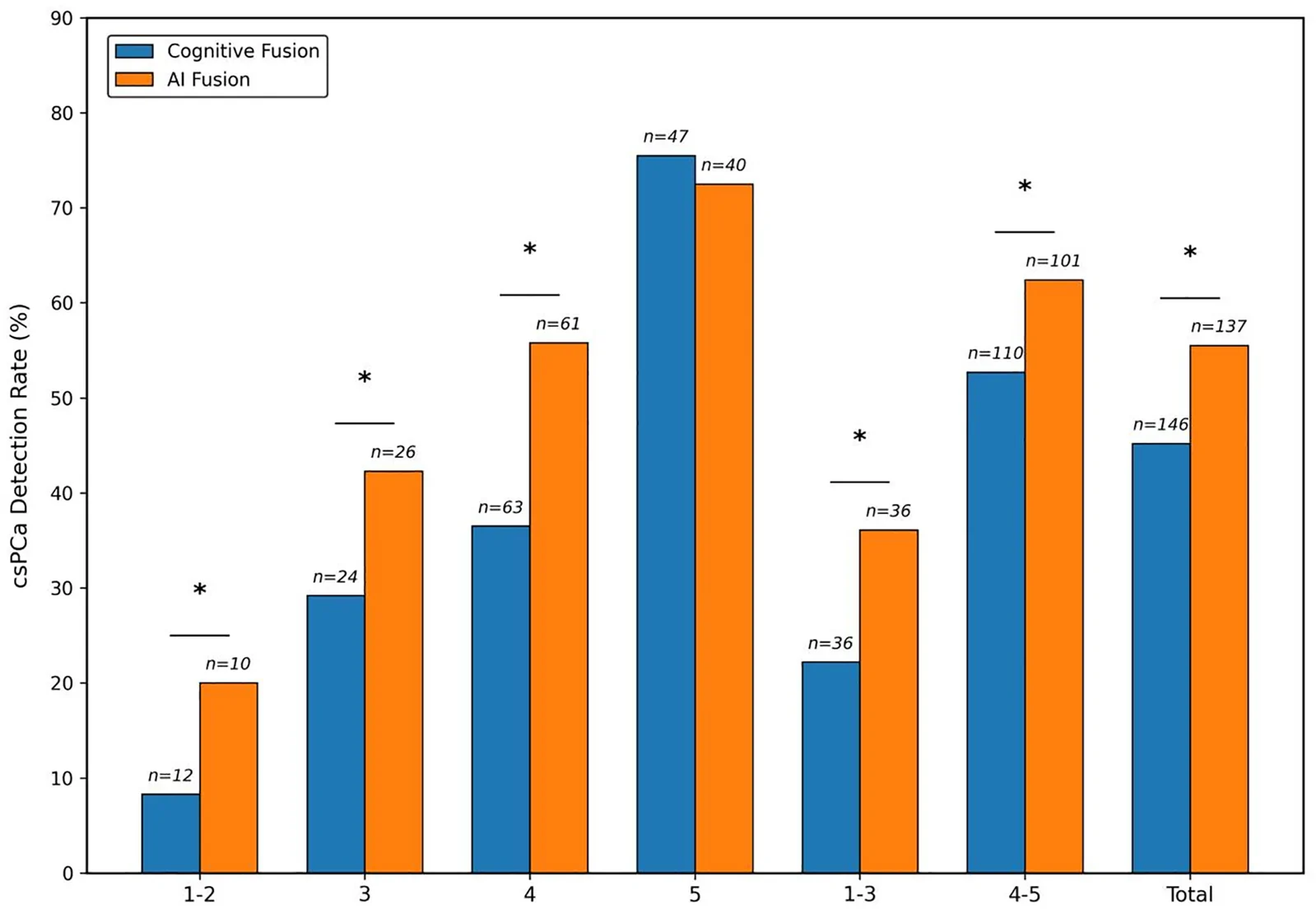
Comparative csPCa detection rates stratified by PI-RADS categories. *P < .05. csPCa = clinically significant prostate cancer, PI-RADS = Prostate Imaging Reporting and Data System.

The AI-navigated fusion biopsy demonstrated a higher overall detection of clinically significant prostate cancer (csPCa), achieving a 55.5% detection rate compared to 45.2% with cognitive fusion (*P* = .043; Fig. 4). Stratified analyses revealed 3 key patterns: Progressive improvement in lower-risk categories (PI-RADS 1–4), with detection rate increases ranging from +11.7% (PI-RADS 1–2) to +19.3% (PI-RADS 4), all statistically significant (*P* < .05); comparable performance for definitive malignancies (PI-RADS 5: 75.5% vs 72.5%, *P* = .637); and consistent clinical utility across risk strata, showing significant gains in both low-intermediate (PI-RADS 1–3: +13.9%, *P* = .045) and high-risk subgroups (PI-RADS 4–5: +9.7%, *P* = .046). Subgroup analyses across individual PI-RADS categories were exploratory; findings in smaller subgroups (e.g., PI-RADS 1–2) should be interpreted with caution due to limited sample sizes.

### 3.3. Univariable and multivariable logistic regression analyses for csPCa detection

Univariable regression analysis identified age (OR = 1.06, 95% CI: 1.02–1.10), PI-RADS score > 3 (OR = 7.29, 95% CI: 2.52–14.36), transperineal approach (OR = 3.74, 95% CI: 1.55–5.27), and anterior target lesions (OR = 3.11, 95% CI: 1.09–4.14) as significant predictors of csPCa (Table 2). Prior biopsy status was not a significant predictor of csPCa detection in univariable analysis (*P* = .283) and was therefore not entered into the multivariable model. Subsequent multivariable logistic regression (dependent variable: csPCa status [1/0]) demonstrated that advanced age (OR = 1.05, 95% CI: 1.01–1.17), PI-RADS scores exceeding 3 (OR = 9.4, 95% CI: 3.95–16.74), and transperineal procedural approach (OR = 3.65, 95% CI: 1.22–4.75) independently predicted csPCa detection (*P* < .05). Anterior target lesion location did not retain statistical significance in the multivariable model (OR = 2.81, 95% CI: 0.83–5.97, *P* = .084; Table 2).

**Table 2:** Univariable and multivariable logistic regression analyses for clinically significant prostate cancer (csPCa) detection.

	Univariate			Multivariate		
	OR	95% CI	*P*	OR	95% CI	*P*
Age	1.06	1.02–1.1	.003	1.05	1.01–1.17	.023
PSAD	1.14	1.04–1.32	.163	–	–	–
Core length, mm	0.82	0.72–1.39	.271	–	–	–
Prostate volume	1.55	1.02–2.53	.118	–	–	–
PI-RADS						
≤3	Reference					
>3	7.29	2.52–14.36	.003	9.4	3.95–16.74	.008
Total number of biopsy samples	1.33	1.02–1.39	.274	–	–	–
Target lesion location						
Posterior	Reference					
Anterior	3.11	1.09–4.14	.033	2.81	0.83–5.97	.084
DRE						
Negative	Reference					
Positive	0.65	0.37–1.16	.177	–	–	–
Biopsy direction						
Transperineal	3.74	1.55–5.27	.036	3.65	1.22–4.75	.006
Transrectal	Reference					
Prior biopsy status						
Biopsy-naive	Reference					
Repeat biopsy	2.37	1.35–4.68	.283	–	–	–

CI = confidence interval, csPCa = clinically significant prostate cancer, DRE = digital rectal examination, OR = odds ratio, PI-RADS = Prostate Imaging Reporting and Data System, PSAD = prostate specific antigen density.

### 3.4. Postoperative complications

**Figure 5.**
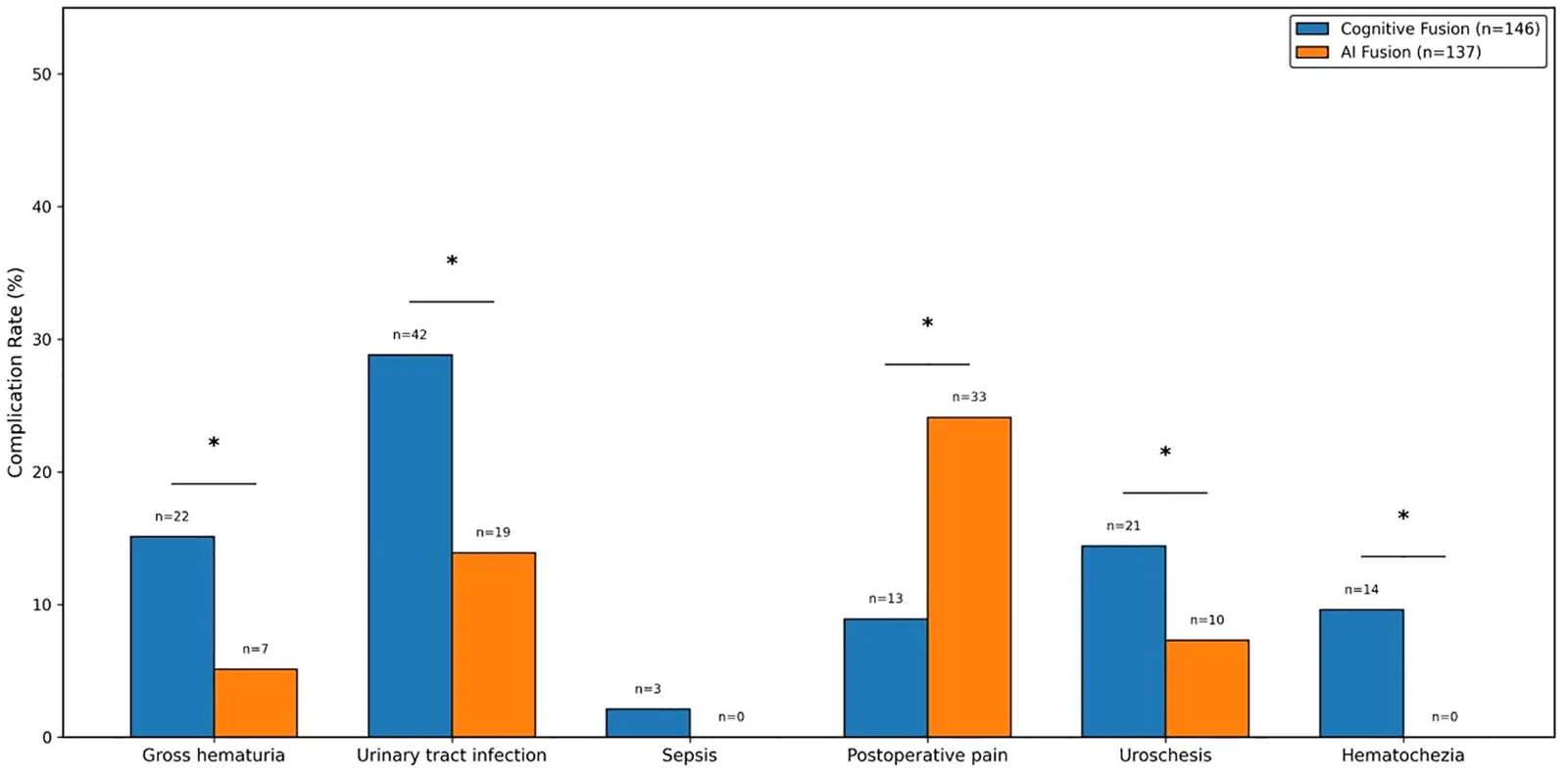
Postoperative complications – n (%). *P < .05. AI = artificial intelligence.

Postoperative complications are summarized in Figure 5. The AI-navigated transperineal group showed a lower rate of urinary tract infections compared to the cognitive fusion group (13.9% vs 28.8%, *P* = .035). Three cases of sepsis occurred, all in the transrectal cognitive fusion group (2.1%), while no sepsis was observed in the AI group (0%); however, this difference was not statistically significant (*P* = .246) given the low event rate. Similarly, the transperineal approach was associated with significantly less hematochezia (0% vs 9.6%, *P* = .003) and gross hematuria (5.1% vs 15.1%, *P* = .025), with all hematuria cases being mild and self-limiting. Urinary retention was significantly more frequent in the transperineal group (14.4% vs 7.3%, *P* = .012), but all cases resolved within 72 hours with α-blocker therapy. Transient perineal pain was also more frequent in the transperineal group (24.1% vs 8.9%, *P* = .017). Notably, 1 patient in the cognitive fusion group required antibiotic escalation to piperacillin-tazobactam for persistent *Escherichia coli* bacteremia, highlighting the infective risks inherent to the transrectal approach. Overall, these findings indicate that the AI-navigated transperineal technique offers a more favorable infectious complication profile, though it carries a higher risk of transient, self-limiting urinary retention.

## 4. Discussion

The increasing incidence of prostate cancer has necessitated advancements in biopsy techniques to enhance detection accuracy, with combined systematic and targeted biopsy (SB + TB) demonstrating improved diagnostic performance over traditional SB alone, as evidenced by multiple comparative studies.^[7-9,18]^ Current targeted biopsy modalities include 3 primary approaches: mpMRI-direct guidance, limited by high costs and specialized equipment requirements^[19]^; *cognitive fusion*, which relies on preoperative mpMRI annotation and intraoperative mental co-registration of TRUS images, requiring substantial operator expertise for spatial reasoning and pattern recognition. While cognitive fusion-based SB + TB improves detection rates, its subjective nature prevents objective validation of MRI-target alignment^[20]^; and *AI fusion*, integrating multimodal artificial intelligence for automated lesion segmentation on mpMRI to generate 3D targets. Real-time fusion of biplane TRUS and mpMRI constructs a dynamic 3D prostate model (Fig. 1), with AI-driven needle tracking providing 2D-3D spatial feedback and optimized trajectory guidance. This method synergizes mpMRI’s excellent soft-tissue contrast with TRUS’s real-time capability, offering a more intuitive navigation system and a shorter learning curve compared to cognitive fusion.

This study retrospectively analyzed 283 patients undergoing transrectal cognitive fusion (n = 146) or transperineal AI fusion biopsy (n = 137) with combined SB + TB protocols. The transperineal AI group demonstrated higher overall csPCa detection rates compared to the transrectal cognitive group (55.5% vs 45.2%, *P* = .043), consistent with Valerio et al.‘s findings (50.5% vs 43.4%, *P* > .05).^[21]^ However, discrepancies exist across studies: El-Achkar et al reported comparable sensitivity/specificity (55% vs 53%) between approaches in 145 patients, with fewer cores and lower complications in the transperineal cohort,^[20]^ while Wu et al’s meta-analysis (n = 8826) confirmed enhanced csPCa detection, particularly for anterior lesions, via the transperineal route.^[22]^ The observed advantage of AI-enhanced transperineal biopsy may stem from 2 factors: Anatomical accessibility enabling comprehensive anterior/apical lesion sampling^[23]^ and AI-driven navigation that automates MRI-US fusion, reduces operator-dependent variability, and optimizes needle trajectory through real-time 3D tracking (Fig. 2), thereby improving spatial accuracy and procedural consistency.

Underdiagnosis of subtle prostate cancer lesions (PI-RADS 1–3) remains a persistent challenge in clinical practice,^[24]^ where manual segmentation of mpMRI-visible lesions – particularly satellite microlesions (<0.5 cm^3^) – is complicated by histopathological heterogeneity (predominantly benign glands and stroma), often leading to volumetric underestimation compared to histopathological references.^[25,26]^ Cognitive fusion biopsies, relying on operator-dependent freehand techniques guided by subjective imaging interpretation, demand exceptional expertise and risk sampling inaccuracies.^[27]^ In contrast, AI-navigated systems enhance precision through automated lesion segmentation and quantitative spatial registration. Supporting this, Fletcher et al reported significantly higher satellite lesion detection rates with AI fusion compared to cognitive approaches,^[28]^ paralleling our findings: the AI fusion group demonstrated higher csPCa detection for PI-RADS 1 to 4 lesions (*P* < .05), while comparable rates were observed in PI-RADS 5 cases (75.5% vs 72.5%, *P* = .637).

Regarding complications, the transperineal AI-navigated group demonstrated a significantly lower rate of urinary tract infections compared to the transrectal cognitive fusion group (13.9% vs 28.8%, *P* = .035). Three cases of sepsis occurred, all in the transrectal cognitive fusion group (2.1%), while none were observed in the transperineal group (0%). However, this difference was not statistically significant (*P* = .246), likely reflecting the limited sample size and low event rate. Hematochezia and gross hematuria were also significantly less frequent in the transperineal group (0% vs 9.6%, *P* = .003; 5.1% vs 15.1%, *P* = .025), attributable to the avoidance of rectal wall puncture. Conversely, urinary retention occurred significantly more often in the transperineal group (14.4% vs 7.3%, *P* = .012). Potential mechanisms include prostatic edema and elevated intraprostatic pressure secondary to transperineal needle trajectories, as well as transient neuropraxia of pelvic floor innervation during perineal access.^[29,30]^ Reassuringly, all retention cases resolved within 72 hours with α-blocker therapy. Transient perineal pain was also more frequent in the transperineal group (24.1% vs 8.9%, *P* = .017), reflecting the access route. Overall, these findings suggest that the AI-navigated transperineal technique is associated with a more favorable infectious complication profile, but at the cost of a significant increase in transient urinary retention. Larger multicenter trials are needed to validate these safety profiles and to explore strategies for mitigating post-biopsy retention.

## 5. Limitations

Several limitations of this study should be acknowledged. First, the retrospective, non-randomized design introduces potential selection bias and confounding. The allocation to biopsy approach was influenced by clinical factors and temporal trends in institutional practice, making it difficult to isolate the independent effect of AI guidance from the transperineal approach. Second, although we adjusted for age, PI-RADS score, prior biopsy status, and lesion location in multivariable analysis, residual confounding from unmeasured variables (e.g., operator experience, number of targeted cores per lesion) cannot be excluded. Third, the exclusion of patients due to incomplete data may introduce selection bias and affect generalizability. Fourth, the study was underpowered for rare complication outcomes such as sepsis. Finally, the single-center design limits external validity. Future multicenter prospective trials are warranted to validate these findings and to disentangle the relative contributions of AI guidance and biopsy approach.

## 6. Conclusions

In this single-center retrospective cohort study, AI-navigated transperineal fusion biopsy was associated with a higher detection rate of clinically significant prostate cancer and a lower incidence of infectious complications compared to transrectal cognitive fusion biopsy, albeit with a significantly higher rate of transient urinary retention that resolved conservatively in all cases. These findings suggest a favorable overall risk-benefit profile, while underscoring the need for patient counseling regarding voiding function. Prospective multicenter studies are warranted to validate these observations and to isolate the independent contributions of AI guidance and biopsy approach.

## Acknowledgments

The authors thank Xin Chen for assistance with data investigation.

## Author contributions

**Conceptualization:** Jiang Chuan Chen.

**Data curation:** Changlong Li.

**Formal analysis:** Qiao Xu.

**Methodology:** Qiao Xu.

**Project administration:** Qiao Xu.

**Software:** Changlong Li.

**Visualization:** Changlong Li.

**Writing – original draft:** Jiang Chuan Chen.

**Writing – review & editing:** Jiang Chuan Chen, Jiamo Zhang.
